# Burden of Mitral Regurgitation in Spain from 2016–2021: An Analysis by Aetiology and Sex

**DOI:** 10.3390/jcm13216372

**Published:** 2024-10-24

**Authors:** José Luis Zamorano, Mercedes Álvarez-Bartolomé, Dabit Arzamendi, Manuel Carnero-Alcázar, Ignacio Cruz-González, Chi-Hion Pedro Li, Ana Pardo-Sanz, Óscar Martínez-Pérez, Mónica Cerezales, Jesús Cuervo, Matteo Vernia, Paloma González, Belén Martí-Sánchez

**Affiliations:** 1 Centro de Investigación Biomédica en Red Enfermedades Cardiovasculares (CIBERCV), 28029 Madrid, Spain; 2 Cardiology Department, University Hospital Ramon y Cajal, 28034 Madrid, Spain; 3 University Hospital Gregorio Marañón, 28009 Madrid, Spain; 4 Cardiology Department, The Thorax Institute, Hospital Clínic, University of Barcelona, 08036 Barcelona, Spain; 5 Department of Cardiac Surgery, Hospital Clínico San Carlos, 28040 Madrid, Spain; 6 Interventional Cardiology, University Hospital of Salamanca, 37008 Salamanca, Spain; 7 Instituto de Investigación Biomédica de Salamanca (IBSAL), 37008 Salamanca, Spain; 8 Cardiology Department, Hospital de la Santa Creu i Sant Pau, 08036 Barcelona, Spain; 9 Biomedical Research Institute (IIB Sant Pau), 08036 Barcelona, Spain; 10 Medicine Department, Universitat Autònoma de Barcelona, 08036 Barcelona, Spain; 11 Faculty of Medicine and Health Sciences, University of Alcalá de Henares, 28034 Madrid, Spain; 12 Axentiva Solutions, 08036 Barcelona, Spain; 13 Applied Economics and Quantitative Methods, University of La Laguna, 38200 Tenerife, Spain; 14 Axentiva Solutions, 33006 Oviedo, Spain; mcerezales@axentiva.com (M.C.); jcuervo@axentiva.com (J.C.); 15 Edwards Lifesciences Europe, 1260 Nyon, Switzerland; matteo_vernia@edwards.com (M.V.); belen_marti@edwards.com (B.M.-S.); 16 Edwards Lifesciences, 28034 Madrid, Spain; paloma_gonzalez@edwards.com

**Keywords:** mitral regurgitation, burden of illness, TEER, sex

## Abstract

**Objectives**: Mitral regurgitation (MR) is the second most common valve disease in Europe, and differences between men and women have been described in relation to aetiology or management, which might impact the decision for intervention and patients’ clinical and economic outcomes. Thus, the objective was to analyse the burden of MR in Spain by aetiology and sex, and the management of all patients suffering from MR being admitted to hospital between 2016–2021. **Methods**: An analysis was carried out with the Ministry of Health’s database, including all patients in public and subsidised hospitals and defining two groups, general MR and those patients undergoing Transcatheter Edge-to-Edge repair (TEER), using a descriptive analysis of patients’ characteristics, use of resources, and outcomes; standardised rates were calculated and observed outcomes were described. **Results**: Hospital admissions increased from 2016 (*n* = 32,806) to 2021 (total *n* = 61,036). In general, the women were older and presented more complications. The majority of patients suffered from degenerative MR (DMR) (*n* = 183,005, 59.55%), and 61.56% were women, contrary to functional MR (FMR) (*n* = 124,278), which consisted of 62.15% males. In total, 1,689 TEERs were performed, 23.33% of them in urgent admissions, and mostly in men (65.66%). All groups showed higher rates of intervention for males. Regarding costs, women presented lower mean costs in the general MR groups but those undergoing TEER presented, in all cases, costs higher than men. **Conclusions**: MR entails a significant burden for patients and the Spanish healthcare system, increasing over the period of study. Differences in aetiologies by sex have been found in patients’ characteristics as well as outcomes. Further studies are needed to optimise patients’ management and their outcomes in relation to sex and aetiology.

## 1. Introduction

Among mitral valve diseases (MVD), mitral regurgitation (MR) is the second most common in Europe [1] and the most prevalent worldwide [2]. Along with other valvular diseases (VDs), its prevalence is rising mostly due to the ageing population and prolonged life expectancy [2]. A recent study in 19 European centres identified a degree of MR in 24% of the patients analysed [3], with 15.5% moderate cases and 5.9% severe cases. Out of these moderate-to-severe patients, primary MR aetiology was detected in 55.1% (with degenerative (DMR) in 59.8% of cases), secondary or functional MR (FMR) in 30.1%, and 14.1% presented a mixed aetiology. Within FMR, two main types can be described based on cardiac morphology and function: atrial and ventricular FMR. Atrial FMR (aFMR) is mostly explained by left atrial dilation in patients with atrial fibrillation (AF), whereas ventricular FMR (vFMR) is secondary to left ventricular dilatation and subsequent annular enlargement [4,5]. Worse prognoses and outcomes (heart failure hospitalisation, freedom from reoperation, all-cause mortality) have been recently described for patients suffering from vFMR, as well as several baseline characteristics in terms of anatomy and functionality [5,6]. Due to the diversity of the disease aetiology, as well as the different management of patients, significant differences have been found between women and men with MR. For example, women have been described to be older and exhibit higher rates of severe calcification, while men usually present more severe left ventricle dysfunction (ejection fraction < 30%) [3]. Differences between sexes have also been reported in terms of disease management, as there is delayed intervention and a disproportionately higher number of replacements in women [7]. In Spain, the Cardiovascular Health Strategy of the National Healthcare System (Estrategia en Salud Cardiovascular del Sistema Nacional de Salud (ESCAV)) includes both sex and gender as factors that should be accounted for when addressing any cardiovascular disease, from detection and diagnoses to treatment [8]. The ESCAV also states that there is a lack of data segregated by sex in the different cardiovascular pathologies and that, although there is increasing evidence highlighting the role of sex and gender in these patients, this can sometimes be crossed over, and healthcare professionals are still underestimating this effect [8].

In general terms, surgical treatment is indicated for both primary and secondary severe MR, but patients with high surgical risk, comorbidities, and favourable anatomy are operated on by transcatheter edge-to-edge repair (TEER). In a French nationwide study, TEER for severe MR seemed to have safer outcomes than surgery in some of the outcomes assessed, such as pacemaker implantation, stroke incidence, and long-term mortality [9], even though further research is required. Although treatment algorithms for MR management are included in the most recent clinical guidelines [1,10] and a consensus document about diagnosis and management in Spain has been released with the participation of the Section on Valvular Disease, Cardiovascular Imaging, Clinical Cardiology, and Interventional Cardiology Associations of the Spanish Society of Cardiology [11], all the aetiologies are still underdiagnosed and undertreated [3,12]. Patients suffering from MR show a high rate of comorbidity and mortality and entail a large burden for national healthcare systems (NHSs) in economic terms [13].

MR remains a highly prevalent disease with important implications both for patients and NHSs. Additionally, there is an urgent need to further improve aspects such as raising awareness, achieving an early diagnosis, and optimised and personalised treatment, given that MR management is still suboptimal in Spain [12].

As there is a lack of knowledge of the real situation of MR in Spain, and specifically by sex, the objective of this work, aligned with the ESCAV, was to analyse the national burden of MR and the in-hospital management of these patients by using the Ministry of Health’s mandatory database, including all patients admitted to hospital with an MR diagnosis during the period 2016–2021 by sex and accounting for MR aetiology.

## 2. Materials and Methods

### 2.1. Data Source

Data were obtained from the Registry of Specialised Healthcare Activity (RAE-CMBD) for 2016–2021 using the coding system from the International Classification of Diseases, tenth revision (ICD-10). Since the administrative database is fully anonymised and no individual patient records are accessed, ethical approval from the Committee is not required. Anonymisation is conducted by the Ministry in accordance with Royal Decree 69/2015, of February 6, which regulates the Registry of Specialised Healthcare Activity. Furthermore, it adheres to the provisions of Organic Law 3/2018, of December 5, on the Protection of Personal Data and Guarantee of Digital Rights, in alignment with the General Data Protection Regulation of the European Union. All available hospitalisations of patients aged 18–99 years, with a diagnosis of MR (main or secondary) at discharge at a public or subsidised hospital, were included in the analysis. MR was categorised by aetiology using a proxy to classify all the cases according to ICD-10 (I34.0). In line with this study’s objectives, all available records were included to facilitate a thorough evaluation of the MR burden on the healthcare system, while accounting for factors beyond the patients’ clinical characteristics. Exclusion criteria were patients suffering from infectious endocarditis, rheumatic mitral disease, and interatrial septal closure procedures (with a total of 2516 patients being excluded from the initial sample). Additionally, the database was screened to identify MR patients undergoing TEER through procedure codes (Table S1). In terms of data management, when multiple records were linked to the same case, episodes were combined (further details on the consolidation process and data handling can be found in Material S1). For these cases, the days of stay, intensive care unit (ICU) stays, outcomes, and complications (Table S2) from all episodes were aggregated and analysed together.

### 2.2. Statistical Approach

A preliminary descriptive analysis of the study variables was performed on the number of patients’ hospitalisations with an MR diagnosis based on age, comorbidities, and complications (Supplemental Table S2), among others. A descriptive analysis of TEER was also carried out. Continuous variables are expressed as mean (standard deviation—SD) and median [interquartile range—IQR], whereas categorical variables are represented in terms of frequency and percentage (%).

Standardised rates (×10^5^ inhabitants) of both diagnoses and procedures were calculated by year, sex, and MR type (DMR or FMR (aFMR or vFMR)), considering the seasonal population (person-year) and sex, which are available at Instituto Nacional de Estadística.

Comorbidities were analysed by calculating the Charlson Comorbidity Index adjusted for age (a-CCI) [14,15], utilizing an algorithm designed for ICD-10 codes and relying on available patient-on-admission (POA) data [16,17].

Furthermore, a descriptive analysis was conducted to examine the burden of illness (BoI) in relation to the length of stay (LoS), in both the ward and ICU, along with the associated costs. LoS was defined as the duration from patient admission to discharge or death.

To estimate the mean annual costs associated with the procedures, all available costs were adjusted to reflect EUR2021 values. The costs recorded in the RAE-CMBD database are determined by the combination of diagnosis-related group (DRG) and severity level [18]. Consequently, the standardization was performed by assigning costs based on the DRG and severity level for the year 2021. For DRG–severity level combinations that lacked a corresponding entry in 2021, costs were updated using the Consumer Price Index.

Regarding the statistical analysis, *t*-tests or ANOVA were employed to examine differences between independent samples of continuous variables. For those that did not follow a normal distribution according to the Anderson–Darling Normality Test, the Mann–Whitney U or Kruskal–Wallis test was also applied. Similarly, Fisher’s exact test was employed to compare independent samples of categorical variables, while the Chi-squared test was utilised when Fisher’s exact test was not suitable. Statistical comparisons were two-tailed and deemed significant if the *p*-value was less than 0.05. Data management and statistical analysis were performed using R software version 4.1.2 (Posit Software, PBC formerly RStudio, PBC. 250 Northern Avenue Suite 420, 02210 Boston, MA, USA).

## 3. Results

### 3.1. Patients’ Hospitalisations with an MR Diagnosis

The total number of hospitalisations with an MR diagnosis was 307,283 for the study period (Table 1). The yearly number of admissions to hospitals with an MR diagnosis increased during the period of study, with 61,036 cases in 2021.

In Table 1 and Table 2, and in various sections of the results, means and SDs, as well as medians and IQRs, are reported to facilitate interpretation of the results and data usability.

The mean aCCI for patients hospitalised with MR was 5.74 (SD 2.45), with 83.33% having an aCCI ≥ 4. The majority of cases were women, with a mean age statistically higher than men. Complications identified in the database were acute myocardial infarction (AMI), atrial fibrillation (AF), acute renal failure (ARF), complications of the prosthesis (Prot), cerebrovascular accidents (Cereb), such as transient ischemic attacks and related syndromes (TIA) and intraoperative cerebrovascular infarction, postprocedural cerebrovascular infarction, stroke resulting from thrombosis of cerebral arteries, stroke caused by emboli in cerebral arteries, cerebral infarction due to unspecified occlusion or stenosis of cerebral arteries (Other), permanent pacemaker implantation (PPI), prolonged intubation requiring tracheostomy (IntubTrac), and endocarditis (Endo). The most prevalent complication was AF (1.16%), followed by PPI (0.62%) and AMI (0.25%). The difference in complication prevalence between sexes can be seen in Table 1 and was found to be statistically significant for six complications.

Regarding BoI, the mean LoS for these patients was 8.97 days (SD 9.82), and the mean ICU LoS was 0.50 days (SD 3.08), with 89.40% of patients admitted to the ICU. Urgent admission was required in 83.59%, while 8.90% died in hospital. Differences in BoI by sex can be seen in Table 1.

The mean cost of all MR patients hospitalised was EUR 6370.05 (SD EUR 7048.90), which increased during the study period from EUR 5553.50 (SD EUR 6120.46) in 2016 to EUR 7860.24 (SD EUR 8336.36) in 2021 (*p*-value < 0.001). Table 1 shows detailed costs by sex.

#### 3.1.1. DMR

Out of all the patients with an MR diagnosis, the total number of hospitalised patients with a DMR diagnosis was 183,005 for the entire period, increasing from 2016 (*n* = 19,991) to 2021 (*n* = 36,255).

Patients with DMR had aCCI values of 5.70 (SD 2.47), with most in the aCCI ≥ 4 group (83.41%). The mean age was 78.56 years (SD 12.02), and more than half the patients were older than 80 years old (54.09%). Females represented 61.56% of the total DMR population.

The differences in complication prevalence between sexes can be seen in Table 1 and were found to be statistically significant for six complications.

Mean DMR LoS was 8.87 days (SD 9.36), and mean ICU LoS was 0.38 days (SD 2.80), with 92.46% of patients admitted to the ICU; men had longer LoS and a higher admission rate to the ICU (Table 1). Urgent admission was required in 83.72% of patients, while 9.03% died in hospital. The mean cost for hospitalisation of patients with DMR was EUR 5885.94 (SD EUR 6396.24), being higher in men (Table 1).

#### 3.1.2. FMR

There were 124,278 hospitalised patients with an FMR diagnosis in the study period, 37.85% of whom were female and 62.15% male, contrary to what was found in DMR patients (61.56% females) (Table 1). The number of cases increased in the period of study from 12,815 in 2016 to 24,781 in 2021.

The mean aCCI was 5.79 (SD 2.43) (Table 1), and the mean age was 75.42 years (SD 11.79). These patients were younger than those suffering from DMR (*p* < 0.001).

The average LoS for these patients was 9.12 days (SD 10.46), and the mean ICU LoS was 0.69 days (SD 3.44), with 84.90% of patients admitted to the ICU. Urgent admission was required for 83.40% of patients, while 8.71% died in hospital. The mean cost of FMR patients’ hospitalisation was EUR 7082.93 (SD EUR 7858.48) and differences by sex can be seen in Table 1.

Results for the different types of FMR (aFMR and vFMR) are presented in the next sections.

##### Atrial FMR

A total of 7394 hospitalised patients with aFMR were found between 2016 and 2021. There was a yearly increase from 762 in 2016 to 1428 in 2021.

In total, 96.35% of patients had an aCCI value ≥ 4, while the mean aCCI was 6.42 (SD 2.02). The mean age was 82.71 years (SD 6.03) and there were no patients younger than 70 years old; 14.53% were 71–75 years old, 21.25% were 76–80 years old, and 64.23% were over 80 years old. Females represented 58.94% of the cases.

Differences between sexes can be seen in Table 1.

The mean LoS was 9.17 days (SD 8.59), and 91.04% of patients were admitted to the ICU with a mean ICU LoS of 0.37 days (SD 2.14). A total 91.28% of these patients had an urgent admission and 12.01% of them died in hospital. The mean cost was EUR 5489.00 (SD EUR 5655.62). Differences by sex can be seen in Table 1.

##### Ventricular FMR

A total of 85.66% (*n* = 106,455) of cases with FMR belonged to vFMR. Total cases rose from 10,837 in 2016 to 21,361 in 2021.

On average, these patients were younger than those suffering from aFMR (mean of 75.09 (SD 11.76), *p* < 0.001), and most of them were male (64.70%). The mean aCCI was 5.79 (SD 2.43), and 83.19% of patients had an aCCI ≥ 4.

The mean LoS was 9.12 days (SD 10.58), and the mean ICU LoS was 0.70 days (SD 3.51), with 84.74% of patients admitted to the ICU. Urgent admission was required in 82.62% of cases, being statistically lower than for aFMR patients along with in-hospital mortality (8.36%). The mean cost of hospitalisation of patients suffering from vFMR was EUR 7226.86 (EUR SD 7935.97), which is higher than for aFMR patients (*p* < 0.001). Differences by sex can be seen in Table 1.

### 3.2. TEER Procedures

Out of the 307,283 patient hospitalisations with an MR diagnosis in the study period, 1689 of them underwent a TEER procedure (Table 2): 595 DMR and 1094 FMR (59 aFMR and 954 vFMR). The total number of TEERs increased during the study period, and 475 of them were performed in 2021. In total, 76.67% of the interventions were elective and mostly performed in men (65.66%).

- DMR: 595 interventions in patients with the following characteristics: mean age 74.20 years (SD 12.31) and mean aCCI of 4.40 (SD 2.05). The mean LoS of these patients for the procedure was 8.37 days (SD 11.13), with 53.54% of patients admitted to the ICU with a mean LoS of 0.68 days (SD 2.41). A total of 13 patients died in hospital (2.18%). The mean hospitalisation cost for DMR patients undergoing TEER was EUR 17,941.91 (SD EUR 8409.22). Complications were balanced between sexes.
- FMR: 1094 hospitalisations undergoing TEER with a mean age of 70.64 years (SD 10.25), which is statistically lower than that of DMR patients undergoing TEER. The mean aCCI was 4.72 (SD 2.00). In total, 73.49% of the cases were male. The mean LoS for these patients was 9.58 days (SD 12.72) and 52.83% required ICU admission with a mean LoS of 1.24 days (SD 5.40). These patients had a mean cost of EUR 17,210.43 (SD EUR 9383.85). There were no statistically significant differences between sexes regarding the prevalence of complications.
  ○ aFMR: 59 TEER procedures were performed. All the patients were older than 71 years old and their mean age was 77.90 years (SD 4.29). In total, 64.41% of them were male and the mean aCCI was 5.10 (SD 2.29). The mean LoS was 10.95 days (SD 13.15), and 22 were admitted to the ICU with a mean LoS of 1.15 days (SD 2.53). There were no deaths. The mean cost of patients suffering from aFMR who underwent a TEER was EUR 18,055.27 (SD EUR 8606.49).
  ○ There were 954 hospitalisations with a vFMR diagnosis undergoing a TEER. The mean age was 70.46 (SD 10.34) and 74.53% were male. The mean aCCI was 4.75 (SD 1.96). Mean LoS was 9.55 days (SD 12.91) and mean ICU LoS was 1.25 days (SD 5.55), with 52.52% requiring an ICU admission. The mean cost of these patients was EUR 17,073.91 (SD EUR 9404.06).

### 3.3. Rates of MR and TEER

**Figure 1:**
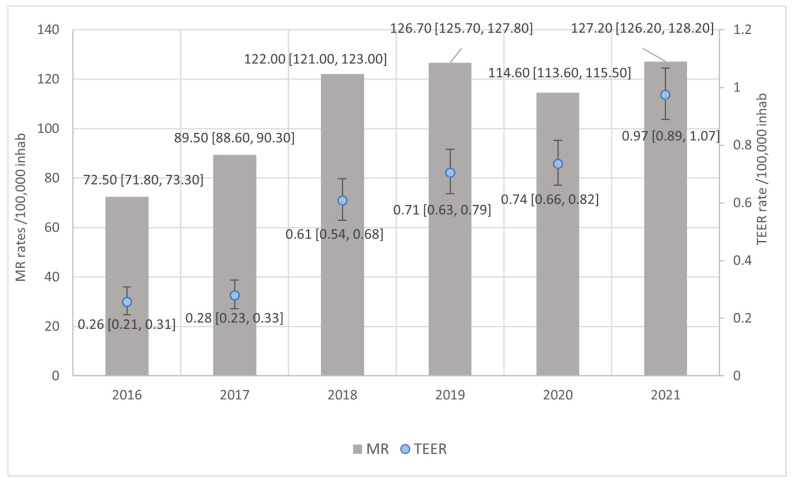
Yearly rates of hospital admissions of patients suffering mitral regurgitation (MR) and Transcatheter Edge-to-Edge repair (TEER) interventions in these patients.

The national utilization rate of TEER for the study period (2016–2021) is 3.76 × 10^5^ inhabitants [95%CI 3.59, 3.95], while the rate of hospital admissions of patients suffering from MR was 655.00 × 10^5^ inhabitants [95%CI 652.00, 657.00]. Both MR and TEER rates showed an increase during the study period (Figure 1).

**Figure 2:**
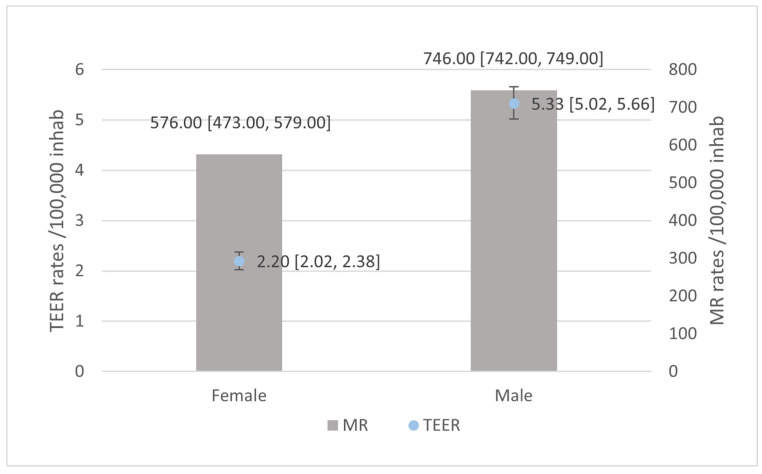
Rates of hospital admissions of patients suffering mitral regurgitation (MR) and patients suffering MR subjected to Transcatheter Edge-to-Edge repair (TEER).

Both MR rates and TEER rates were higher for males than for females (Figure 2) for the entire study period.

**Figure 3:**
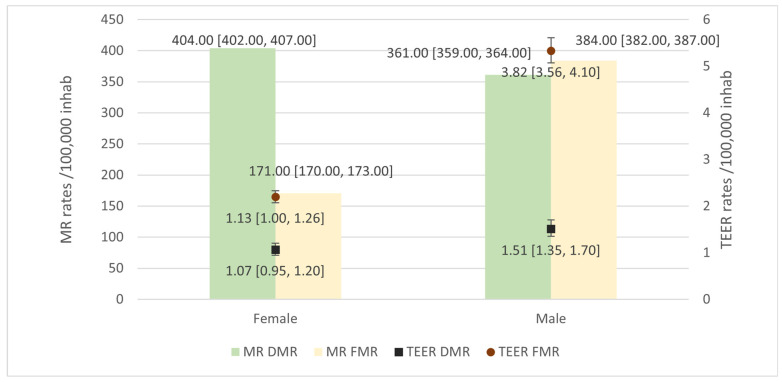
Degenerative mitral regurgitation (DMR) and functional mitral regurgitation (FMR) rates by sex and Transcatheter Edge-to-Edge repair (TEER) rates in those patients.

General DMR rates were higher than FMR rates, at 390.00 × 10^5^ inhabitants [95%CI 388.00–392.00] and 265.00 × 10^5^ inhabitants [95%CI 263.00–266.00], respectively. Patterns by sex showed a higher rate for women in DMR and higher for men in FMR (Figure 3). TEER utilization rates were higher for FMR at 2.33 × 10^5^ inhabitants [95%CI 2.19–2.47] than for DMR at 1.27 × 10^5^ inhabitants [95%CI 1.17–1.37]. Data by sex showed higher rates for men in TEER for both DMR and FMR.

**Figure 4:**
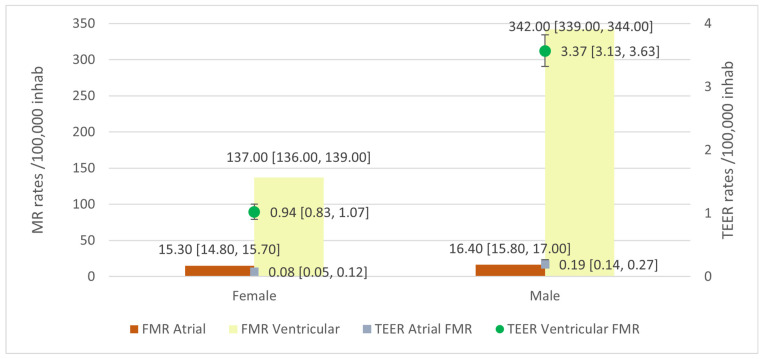
Atrial functional mitral regurgitation (aFMR) and ventricular functional mitral regurgitation (vFMR) rates and Transcatheter Edge-to-Edge repair (TEER) rates in those patients.

vFMR rates were higher than aFMR rates, at 227.00 × 10^5^ inhabitants [95%CI 225.00, 228.00] and 15.80 × 10^5^ inhabitants [95%CI 15.40, 16.10], respectively. This was also seen in TEER rates in these two groups, with rates of TEER 2.03 × 10^5^ inhabitants [95%CI 1.91, 2.17] for vFMR patients and 0.13 × 10^5^ inhabitants [95%CI 0.10, 0.17] for aFMR patients. Rates by sex in vFMR and aFMR for TEER are shown in Figure 4.

## 4. Discussion

This study comprises new detailed evidence regarding the epidemiologic situation of MR in hospitals in Spain, and it is a good estimation of the BoI as it includes all hospitalisations of patients suffering MR in all public and subsidised centres in the country. The distribution of subtypes was aligned with those found in previous studies, being the majority of cases of DMR [3,19], while both sexes were similarly represented in the general MR group. Specific results for subtypes in females were also aligned with previous European research [3,20], with DMR sufferers comprising mostly women (70.54%), while males were equally distributed between DMR and FMR. What was seen in these patients is that men were generally younger and presented lower aCCI scores; this tendency has been previously reported in aortic valvular disease [21], as women are generally diagnosed later than men. These MR patients presented high rates of admission to the ICU, around 90%, which is also higher in women, highlighting the severity and high burden of disease as well as the economic impact on the healthcare system.

During the entire study period, an increase was observed in the rate of patients suffering from MR, with the rate of the last year being double that of the first one. This tendency has been described in other heart valve diseases as a consequence of the ageing population, as well as an improvement in clinical awareness following the development of therapeutic technologies [12]. One of the approaches for the treatment of these patients is TEER, which is indicated for patients suffering from DMR who are inoperable or at high surgical risk, as well as symptomatic patients suffering from FMR, who are ineligible for surgery and fulfil criteria that suggest a chance of responding to TEER [1,11]. Half the patients undergoing TEER were found to be admitted to the ICU, with low rates of urgent admission to hospital and death, which has already been reported in previous experiences in our country when analysing small samples of patients subjected to TEER [22]. Mortality rates up to hospital discharge are also supported by data from the American College of Cardiology/Society of Thoracic Surgeons Transcatheter Valve Therapy registry and the Society of Thoracic Surgeons adult cardiac surgery database, which found a 3.5% mortality at 30 days for patients undergoing TEER [23]. These mortality results are also in accordance with an analysis of 558 patients subjected to transcatheter mitral valve repair in our country, which reported a mortality of around 3% up to hospital discharge [24]. The fact that there was a low rate of urgent admissions is of relevance since it could allow for better resource allocation and thus an optimised process for other patients. A recent study carried out in Switzerland and including all consecutive patients undergoing TEER between 2011 and 2018 found similar results to what has been found in our research in terms of baseline characteristics, where women were, in general, older and had similar rates of comorbidities [20]. However, the results of our research highlight that women in Spain are subjected to more urgent procedures and that their mortality rates are higher than for men, contrary to what was detected in their Swiss counterparts [20]. In this sample, a very low rate of TEER was identified, although an increase was also observed during the study period; these low rates were specially marked for DMR patients. Within DMR patients undergoing TEER, women have been reported to be referred late for intervention, which reduces access to life-saving interventions, leading to worse outcomes when compared to males [25]. Intervention rates for other valves such as the aortic have been reported to be 33 × 10^5^ inhabitants in our country, giving a context for the low intervention rates found in patients suffering from MR [26]. Intervention rates were always lower for females, which might be partly explained by suboptimal management of females, as has been reported elsewhere [7]. Several differences have been described between the sexes regarding MR aetiology and diagnosis, which influences the type and time of intervention [7]. For example, the necessity of having more women be included in research populating the evidence for the establishment of updated diagnosis indexes and thresholds has been recently published, as current measures are mostly adapted to men [25]. This might lead to improved and timely diagnoses for women and a better referral rate. These differences should be considered when considering intervention as this might have implications for improving patient outcomes [21]. For example, in our study, females suffering from MR presented higher rates of mortality and urgent admission even though costs were significantly lower in all cases. Females were older in all the MR groups and the TEER groups [27,28]. In addition to being older, other studies have reported that women are treated later [27], which could lead to worse outcomes, which was also seen in our research in the general MR group but not in those undergoing TEER. This fact could be due to better management of those women subjected to TEER, maybe because of its novelty and the level of awareness of clinicians when performing this technique. In the comparison of females and males undergoing TEER, we could observe a higher rate of urgent admissions and worse outcomes in terms of LoS (except aFMR) and mortality in women, despite not being statistically significant in all groups; similar results in terms of mortality have been found in a previous study focusing on mitral surgery [27], while most studies conclude that there are non-significant differences in mortality for both mitral surgery and transcatheter procedures [27,29,30], or even that women have been found to have better outcomes in terms of mortality [20]. These worse outcomes in women could be mediated by more challenging repairs due to more unfavourable valve anatomy caused by greater calcifications and degenerative pathology [28,30], possibly due to their older age. However, it is known that sometimes factors that are attributed to sex are mediated by a sex–gender interaction that needs to be carefully revisited [8]. All these clinical factors have a direct implication on hospitalisation costs, which have always been found to be higher for women, although this is not always statistically significant in all groups of MR patients undergoing TEER, contrary to what was seen in the groups of patients suffering from MR who were admitted to hospital. Some institutional changes are warranted to improve the situation and ensure equal access and equal outcomes for both women and men, including sex in the personalised medicine scheme currently spreading. Some changes that would be feasible in the Spanish setting could include an optimised diagnosis based on sex-adjusted imaging studies, including body size in the indexes for referral, addressing psycho-social barriers, and others that would end up in official Societal Guidelines updates considering all these sex-specific items.

MR has been reported to entail a significant economic burden to healthcare systems as these patients have more comorbidities and higher mortality than the general population [13]. The results of our study show higher costs for those patients undergoing TEER in comparison with the general group, but these costs seem to be offset in the long term [13].

In this study, all patients with an MR diagnosis admitted to the hospital were included, and thus, final figures must slightly differ from real national numbers as those who did not have a hospital admission or those not diagnosed are not accounted for.

### Limitations

First, although we used the widest national data source—a standardised, validated, and mandatory database representing national hospital activity (for both public and subsidised centres)—the data may still be subject to inherent errors. This potential for inaccuracies arises from possible coding errors or omissions in the recorded information, which could undermine the precision of the results. To alleviate potential issues related to coding inaccuracies, specific measures were taken, including the consolidation of consecutive episodes, aimed at minimising adverse effects resulting from the coding process. Additionally, it is important to note that comorbidities were not directly extracted from medical records; rather, they were derived from the specific diagnoses present upon admission for each hospital stay. Consequently, if any of these diagnoses were not coded, the calculated aCCI would yield a lower value than the actual one. Additionally, sub-aetiologies/subgroups of DMR could not be identified in the database due to the nature of the codification system.

Second, our analysis is based on RAE-CMBD data that restrict information to in-hospital stays and up to hospital discharges. As a result, we are constrained in assessing other significant factors of interest, such as complications after discharge, resources employed during diagnosis, or ambulatory follow-up.

Thirdly, certain factors should be taken into account when interpreting the results and this analysis. It is recognised that the significance of *p*-values is affected by a substantial increase in sample size, which may lead to distortions in the inferences drawn.

Fourthly, in this study, we have not analysed the burden and costs associated with mitral surgery, which remains the most frequently used therapy for patients with severe MR. This may constitute a limitation when interpreting the findings of this study.

Finally, it should be noted that the study period includes the years of the COVID-19 pandemic. The impact of the pandemic on the healthcare system may significantly affect the health outcomes of these years.

## Figures and Tables

**Table 1:** Characteristics and complications of patients suffering mitral regurgitation (MR) admitted to a Spanish public or subsidised hospital for any cause in the period 2016–2021 by sex and MR type.

	Level	MR			MR Type											
		Total			DMR			FMR								
					Total			Total				Atrial		Ventricular		
		F	M		F	M		F	M		F	M		F	M	
*n*		159,686	147,597		112,650	70,355		47,036	77,242		4358	3036		37,583	68,872	
Age (mean (SD))		79.76 (11.29)	74.62 (12.23)	***	80.23 (11.32)	75.89 (12.61)	***	78.64 (11.14)	73.46 (11.74)	***	83.44 (5.91)	81.66 (6.06)	***	78.34 (11.21)	73.31 (11.66)	***
Age (median [IQR])		82.00 [75.00, 87.00]	77.00 [67.00, 84.00]	***	83.00 [76.00, 88.00]	79.00 [69.00, 85.00]	***	81.00 [73.00, 86.00]	75.00 [66.00, 83.00]	***	84.00 [79.00, 88.00]	82.00 [77.00, 86.00]	***	81.00 [73.00, 86.00]	75.00 [66.00, 82.00]	***
Charlson (mean (SD))		5.80 (2.29)	5.67 (2.62)	***	5.74 (2.31)	5.63 (2.71)	***	5.93 (2.24)	5.70 (2.53)	***	6.32 (1.90)	6.58 (2.17)	***	5.95 (2.23)	5.71 (2.52)	***
Charlson (median [IQR])		6.00 [4.00, 7.00]	5.00 [4.00, 7.00]	***	6.00 [4.00, 7.00]	5.00 [4.00, 7.00]	***	6.00 [4.00, 7.00]	6.00 [4.00, 7.00]	***	6.00 [5.00, 7.00]	6.00 [5.00, 8.00]	***	6.00 [4.00, 7.00]	6.00 [4.00, 7.00]	***
FA, *n* (%)		1605 (1.01)	1,954 (1.32)	***	993 (0.88)	861 (1.22)	***	612 (1.30)	1,093 (1.42)	***	76 (1.74)	69 (2.27)		516 (1.37)	983 (1.43)	
AMI, *n* (%)		309 (0.19)	447 (0.30)	***	2 (0.00)	0 (0.00)		307 (0.65)	447 (0.58)	•	60 (1.38)	41 (1.35)		167 (0.44)	313 (0.45)	
Prot, *n* (%)		117 (0.07)	181 (0.12)	***	63 (0.06)	67 (0.10)	**	54 (0.11)	114 (0.15)		2 (0.05)	2 (0.07)		49 (0.13)	102 (0.15)	
Cereb, *n* (%)	TIA	110 (0.07)	81 (0.05)	•	67 (0.06)	36 (0.05)		43 (0.09)	45 (0.06)		4 (0.09)	1 (0.03)		37 (0.10)	42 (0.06)	•
	Other	260 (0.16)	275 (0.19)		151 (0.13)	111 (0.16)		109 (0.23)	164 (0.21)	•	7 (0.16)	4 (0.13)		85 (0.23)	145 (0.21)	
ARF, *n* (%)		158 (0.10)	258 (0.17)	***	92 (0.08)	99 (0.14)	***	66 (0.14)	159 (0.21)		1 (0.02)	7 (0.23)	*	53 (0.14)	135 (0.20)	*
PPI, *n* (%)		892 (0.56)	999 (0.68)	***	591 (0.52)	439 (0.62)	**	301 (0.64)	560 (0.72)	*	21 (0.48)	14 (0.46)		244 (0.65)	502 (0.73)	
IntubTraq, *n* (%)		227 (0.14)	406 (0.28)	***	150 (0.13)	190 (0.27)	***	77 (0.16)	216 (0.28)	•	0 (0.00)	11 (0.36)	***	65 (0.17)	187 (0.27)	**
Endo, *n* (%)		296 (0.19)	328 (0.22)	*	213 (0.19)	211 (0.30)	***	83 (0.18)	117 (0.15)	***	12 (0.28)	8 (0.26)		53 (0.14)	88 (0.13)	
LoS -days- (mean (SD))		8.78 (8.99)	9.17 (10.64)	***	8.72 (8.85)	9.12 (10.12)	***	8.95 (9.33)	9.22 (11.10)		8.92 (7.42)	9.52 (10.03)	**	8.96 (9.46)	9.21 (11.15)	***
LoS -days- (median [IQR])		7.00 [4.00, 11.00]	7.00 [4.00, 11.00]	•	7.00 [4.00, 11.00]	7.00 [4.00, 11.00]	*	7.00 [4.00, 11.00]	7.00 [4.00, 11.00]		7.00 [4.00, 11.00]	7.00 [4.00, 11.00]		7.00 [4.00, 11.00]	7.00 [4.00, 11.00]	
ICU, *n* (%)		146,366 (91.86)	127,750 (86.75)	***	105,426 (93.77)	63,454 (90.37)	***	40,940 (87.28)	64,296 (83.45)	***	4024 (92.51)	2693 (88.94)	***	32,598 (86.99)	57,363 (83.51)	***
ICU LoS -days- (mean (SD))		0.38 (2.69)	0.64 (3.44)	***	0.30 (2.49)	0.50 (3.22)	***	0.55 (3.11)	0.77 (3.62)	***	0.24 (1.47)	0.55 (2.83)	***	0.57 (3.23)	0.77 (3.65)	***
ICU LoS -days- (median [IQR])		0.00 [0.00, 0.00]	0.00 [0.00, 0.00]	***	0.00 [0.00, 0.00]	0.00 [0.00, 0.00]	***	0.00 [0.00, 0.00]	0.00 [0.00, 0.00]	***	0.00 [0.00, 0.00]	0.00 [0.00, 0.00]	***	0.00 [0.00, 0.00]	0.00 [0.00, 0.00]	***
Death, *n* (%)		14,777 (9.25)	12,560 (8.51)	***	10,330 (9.17)	6187 (8.79)	**	4447 (9.45)	6373 (8.25)	***	486 (11.15)	402 (13.24)	**	3434 (9.14)	5467 (7.94)	***
Urgent, *n* (%)		137,498 (86.11)	119,365 (80.87)	***	96,712 (85.85)	56,497 (80.30)	***	40,786 (86.71)	62,868 (81.39)	***	4053 (93.00)	2696 (88.80)	***	32,217 (85.72)	55,735 (80.93)	***
Cost (mean (SD))		5821.89 (6152.99)	6963.11 (7861.74)	***	5545.49 (5748.42)	6431.05 (7281.98)	***	6483.85 (6983.60)	7447.73 (8325.38)	***	5123.02 (4373.58)	6014.34 (7070.13)	***	6681.84 (7149.07)	7524.27 (8319.10)	***
Cost (median [IQR])		3800.06 [3009.44, 5938.61]	4,270.57 [3220.05, 6985.21]	***	3800.06 [3009.44, 5609.57]	3,953.46 [3058.76, 6044.43]	***	4167.33 [3243.38, 6604.27]	4521.54 [3399.29, 7741.21	***	3800.06 [3013.75, 5211.42]	3814.45 [3130.35, 5880.25]	***	4337.93 [3298.43, 6999.38]	4531.38 [3408.79, 8041.93]	***
TEER, *n* (%)		580 (0.36)	1109 (0.75)	***	290 (0.26)	305 (0.43)	***	290 (0.62)	804 (1.04)	***	21 (0.48)	38 (1.25)	***	243 (0.65)	711 (1.03)	***

AF: Atrial Fibrillation. AMI: Acute Myocardial Infarction. ARF: Acute Renal Failure requiring renal function replacement therapy. Cereb: cerebrovascular accidents. DMR: Degenerative mitral regurgitation. Endo: All endocarditis cases. F: Female. FMR: Functional mitral regurgitation. ICU: Intensive care unit. IQR: Interquartile range. IntubTraq: Prolonged intubation requiring tracheostomy. LoS: Length of stay. M: Male. PPI: permanent pacemaker implantation. MR: Mitral regurgitation. Other: Intraoperative cerebrovascular infarction (I97.81*), Postprocedural cerebrovascular infarction (I97.82*), Stroke due to thrombosis of cerebral arteries (I63.3*), Stroke due to emboli of cerebral arteries (I63.4*), Cerebral infarction due to unspecified occlusion or stenosis of cerebral arteries (I63.5*). Prot: Complications of the prosthesis. SD: Standard deviation. TIA: Transient ischemic attacks and related syndromes. TEER: Transcatheter edge-to-edge repair. The t-test or the Mann–Whitney U test is applied to continuous variables based on normality (Anderson–Darling Normality Test), and Fisher’s exact test was applied for categorical variables. Significance levels refer to the comparison of variables between female and male groups. ‘***’ < 0.001, ‘**’ < 0.01, ‘*’ < 0.05, ‘•’ < 0.1, ‘ ’ ≤ 1.

**Table 2:** Characteristics and complications of patients suffering mitral regurgitation (MR) undergoing a Transcatheter Edge-to-Edge repair (TEER) procedure in a Spanish public or subsidised hospital in the period 2016–2021 by sex and MR type.

		MR			MR Type											
		Total			DMR			FMR								
					Total			Total				Atrial		Ventricular		
	Level	F	M		F	M		F	M		F	M		F	M	
*n*		580	1109		290	305		290	804		21	38		243	711	
Age (mean (SD))		73.78 (11.74)	70.90 (10.70)	***	76.25 (11.74)	72.25 (12.53)	***	71.30 (11.22)	70.40 (9.87)		78.14 (4.28)	77.76 (4.34)		70.99 (11.64)	70.28 (9.86)	
Age (median [IQR])		77.00 [68.00, 82.00]	73.00 [65.00, 79.00]	***	80.00 [73.00, 84.00]	75.00 [64.00, 82.00]	***	74.00 [65.00, 80.00]	72.00 [65.00, 77.00]	*	77.00 [75.00, 81.00]	78.00 [74.00, 81.75]		74.00 [65.00, 80.00]	72.00 [64.50, 77.00]	*
Charlson (mean (SD))		4.48 (1.84)	4.67 (2.11)	•	4.42 (1.75)	4.37 (2.30)		4.55 (1.93)	4.78 (2.02)	•	4.95 (1.66)	5.18 (2.60)		4.57 (1.90)	4.81 (1.99)	
Charlson (median [IQR])		4.00 [3.00, 6.00]	4.00 [3.00, 6.00]		4.00 [4.00, 5.00]	4.00 [3.00, 6.00]		4.00 [3.00, 6.00]	5.00 [3.00, 6.00]		4.00 [4.00, 6.00]	5.00 [4.00, 5.75]		4.00 [3.00, 6.00]	5.00 [3.00, 6.00]	
FA, *n* (%)		19 (3.28)	22 (1.98)		8 (2.76)	9 (2.95)		11 (3.79)	13 (1.62)	•	1 (4.76)	3 (7.89)		10 (4.12)	9 (1.27)	*
AMI, *n* (%)		1 (0.17)	2 (0.18)		0 (0.00)	0 (0.00)		1 (0.34)	2 (0.25)		0 (0.00)	1 (2.63)		1 (0.41)	1 (0.14)	
Prot, *n* (%)		7 (1.21)	8 (0.72)		5 (1.72)	1 (0.33)		2 (0.69)	7 (0.87)		0 (0.00)	1 (2.63)		2 (0.82)	5 (0.70)	
Cereb, *n* (%)	TIA	4 (0.69)	1 (0.09)	•	2 (0.69)	0 (0.00)		2 (0.69)	1 (0.12)		0 (0.00)	0 (0.00)		2 (0.82)	1 (0.14)	
	Other	2 (0.34)	4 (0.36)		0 (0.00)	1 (0.33)		2 (0.69)	3 (0.37)		0 (0.00)	0 (0.00)		1 (0.41)	2 (0.28)	
ARF, *n* (%)		5 (0.86)	4 (0.36)		3 (1.03)	0 (0.00)		2 (0.69)	4 (0.50)		0 (0.00)	1 (2.63)		2 (0.82)	3 (0.42)	
PPI, *n* (%)		1 (0.17)	4 (0.36)		4 (1.38)	3 (0.98)		1 (0.34)	3 (0.37)		0 (0.00)	0 (0.00)		1 (0.41)	3 (0.42)	
IntubTraq, *n* (%)		1 (0.17)	4 (0.36)		1 (0.34)	1 (0.33)		0 (0.00)	3 (0.37)		0 (0.00)	0 (0.00)		0 (0.00)	3 (0.42)	
Endo, *n* (%)		0 (0.00)	0 (0.00)		0 (0.00)	0 (0.00)		0 (0.00)	0 (0.00)		0 (0.00)	0 (0.00)		0 (0.00)	0 (0.00)	
LoS -days- (mean (SD))		10.02 (13.49)	8.70 (11.44)	*	9.47 (13.26)	7.32 (8.53)	*	10.57 (13.72)	9.23 (12.32)		8.62 (9.60)	12.24 (14.71)		10.74 (14.39)	9.14 (12.35)	
LoS -days- (median [IQR])		5.00 [3.00, 10.00]	5.00 [3.00, 8.00]		5.00 [3.00, 10.00]	5.00 [3.00, 8.00]		5.50 [3.00, 11.00]	5.00 [3.00, 9.00]	•	5.00 [3.00, 11.00]	5.00 [4.00, 13.75]		6.00 [3.00, 10.00]	5.00 [3.00, 9.00]	•
ICU, *n* (%)		306 (52.76)	590 (53.25)		152 (52.41)	166 (54.61)		154 (53.10)	424 (52.74)		7 (33.33)	15 (39.47)		130 (53.50)	371 (52.18)	
ICU LoS -days- (mean (SD))		0.96 (3.22)	1.09 (5.16)		0.67 (2.02)	0.68 (2.73)		1.26 (4.06)	1.24 (5.81)		0.76 (1.37)	1.37 (2.98)		1.16 (3.53)	1.28 (6.09)	
ICU LoS -days- (median [IQR])		0.00 [0.00, 1.00]	0.00 [0.00, 1.00]		0.00 [0.00, 1.00]	0.00 [0.00, 1.00]		0.00 [0.00, 1.00]	0.00 [0.00, 1.00]		0.00 [0.00, 1.00]	0.00 [0.00, 1.00]		0.00 [0.00, 1.00]	0.00 [0.00, 1.00]	
Death, *n* (%)		18 (3.10)	19 (1.71)	•	8 (2.76)	5 (1.64)		10 (3.45)	14 (1.74)		0 (0.00)	0 (0.00)		8 (3.29)	12 (1.69)	
Urgent, *n* (%)		147 (25.34)	247 (22.27)		71 (24.48)	53 (17.38)	*	76 (26.21)	194 (24.13)		8 (38.10)	11 (28.95)		62 (25.51)	171 (24.05)	
Cost (mean (SD))		18,014.36 (8500.58)	17,182.44 (9325.39)	•	18,089.21 (8603.36)	17,801.85 (8232.10)		17,939.50 (8410.75)	16,947.46 (9702.43)		19,707.61 (9696.67)	17,142.13 (7929.80)		17,853.98 (8362.71)	16,807.31 (9725.61)	
Cost (median [IQR])		17,378.92 [13,044.88, 18,880.02]	17,378.92 [11,994.50, 18,880.02]	**	17,378.92 [13,044.88, 18,880.02]	17,378.92 [13,044.88, 23,229.36]		17,378.92 [13,044.88, 18,880.02]	17,378.92 [11,107.70, 18,880.02]	**	17,378.92 [13,044.88, 18,880.02]	16,610.06 [13,044.88, 18,504.75]		17,378.92 [13,044.88, 18,880.02]	17,378.92 [11,107.70, 18,273.07]	*

AF: Atrial Fibrillation. AMI: Acute Myocardial Infarction. ARF: Acute Renal Failure requiring renal function replacement therapy. Cereb: cerebrovascular accidents. DMR: Degenerative mitral regurgitation. Endo: All endocarditis cases. F: Female. FMR: Functional mitral regurgitation. ICU: Intensive care unit. IQR: Interquartile range. IntubTraq: Prolonged intubation requiring tracheostomy. LoS: Length of stay. M: Male. PPI: permanent pacemaker implantation. MR: Mitral regurgitation. Other: Intraoperative cerebrovascular infarction (I97.81*), Postprocedural cerebrovascular infarction (I97.82*), Stroke due to thrombosis of cerebral arteries (I63.3*), Stroke due to emboli of cerebral arteries (I63.4*), Cerebral infarction due to unspecified occlusion or stenosis of cerebral arteries (I63.5*). Prot: Complications of the prosthesis. SD: Standard deviation. TIA: Transient ischemic attacks and related syndromes. TEER: Transcatheter edge-to-edge repair. The t-test or the Mann–Whitney U test is applied to continuous variables based on normality (Anderson–Darling Normality Test), and Fisher’s exact test was applied for categorical variables. Significance levels refer to the comparison of variables between female and male groups: ‘***’ < 0.001, ‘**’ < 0.01, ‘*’ < 0.05, ‘•’ <0.1, ‘ ’ ≤ 1.

## Data Availability

The data presented in this study are available on request from the Registry of Specialised Healthcare Activity-Minimum Basic Dataset (RAE-CMBD). The data request can be done at https://www.sanidad.gob.es/estadEstudios/estadisticas/cmbdhome.htm.

## Supplementary Materials

The following supporting information can be downloaded at: https://www.mdpi.com/article/10.3390/jcm13216372/s1, Table S1: Procedure groups and ICD10-ES codes; Material S1: Consecutive episodes; Table S2: Complications codes.
