# Supplementary material for: Burden of Mitral Regurgitation in Spain from 2016–2021: An Analysis by Aetiology and Sex
Source: J Clin Med. 2024 Oct 24;13(21):6372. doi: 10.3390/jcm13216372 (PMC11545960; doi:10.3390/jcm13216372)
Supplement: Supplementary file 1 [file jcm-13-06372-s001.zip › jcm-3233138-supplementary.pdf]

Supplementary material:

Table S1. Procedure groups ICD10-ES codes:

Transcatheter edge-to-edge repair: includes all patients with a diagnosis of mitral valve disease who have undergone a transcatheter mitral valve repair	
02QG3ZZ	Procedimiento Médico-Quirúrgico, Corazón y Grandes Vasos, Reparación, Válvula Mitral, percutáneo, sin dispositivo, sin calificador
02UG3JZ	Procedimiento Médico-Quirúrgico, Corazón y Grandes Vasos, Suplemento, Válvula Mitral, percutáneo, sustituto sintético, sin calificador
02QG4ZZ	Procedimiento Médico-Quirúrgico, Corazón y Grandes Vasos, Reparación, Válvula Mitral, endoscópico percutáneo, sin dispositivo, sin calificador

Material S1. Consecutive episodes:

In the case of several records associated with the same case (variable "identification", created from a combination of the variables "recoded CIP SNS" and "date of birth"), the consecutive episodes were consolidated considering the first admission of a patient during the study period as "index event" and the following episodes were concatenated if the discharge date of the first episode coincided with the entry date of the next. To consistently manage the information for the estimation of hospital stays, deaths, groups of procedures, and complications, it was ensured through the validation of the Diagnosis Related Groups (DRG) that these events' consecutive records were not records of duplicate procedures. Otherwise, it was verified that the groups of procedures were the same in the different registries along with the type of discharge such as "transfer to another hospital", or that the dates of procedure in the registries coincided, which would also be indicative of a duplicated registration. In all these cases, LoS, stays in the ICU, exitus variable, and complications of all their episodes were computed jointly. Lastly, for all the cases that did not meet these specifications, all the procedures recorded in subsequent admissions were recorded in the index event, in addition to the imputation of hospital stay and exitus explained above.

Table S2. Complications codes:

The following table shows ICD-10 codes used to identify the complications during hospital stay

ICD-10 complications during hospital stay

Complications	ICD-10 Codes
Atrial fibrillation	I48.0, I48.1, I48.2, I48.91, I48.11, I48.19, I48.20, I48.21
Acute renal failure requiring renal replacement therapy	5A1D00Z,5A1D60Z, N17
Permanent pacemaker implantation	5A1223Z
Prolonged intubation requiring tracheostomy	0B113F4, 0B110F4
Prosthesis complications	T82.0XA, T82.8.7A, T82.9XXA
Endocarditis	I38, I33
Intraoperative complications or post-procedural disorders	I97.88, I97.89, I97.4, I97.6, I97.5, T81.1XA
Acute myocardial infarction	I21
Sepsis and infections	A41, T81.4XXA
Stroke	I97.81, I97.82, I63.3, I63.4, I63.5, G45
